# Unfavourable birth outcomes of the Roma women in the Czech Republic and the potential explanations: a population-based study

**DOI:** 10.1186/1471-2458-5-106

**Published:** 2005-10-10

**Authors:** Martin Bobak, Jan Dejmek, Ivo Solansky, Radim J Sram

**Affiliations:** 1 International Centre for Health and Society, Department of Epidemiology and Public Health, University College London, 1–19 Torrington Place, London WC1E 6BT, UK; 2 Laboratory of Genetic Ecotoxicology, Institute of Experimental Medicine, Videnska 1083, Prague 4, Czech Republic

## Abstract

**Background:**

Data on the health status of the Roma people in Central and Eastern Europe are sparse and the reasons for their poor health are not clear. The objective of this study was to quantify the differences in birth outcomes between Roma and non-Roma mothers in the Czech Republic and to investigate the potential causes of such differences.

**Method:**

A population-based study recruited 8938 non-Roma and 1388 Roma hospitalised singleton births that occurred in two Czech districts (Teplice and Prachatice) between 1995 and 2004. During their stay in hospital, mothers completed a questionnaire on their demographic and socioeconomic characteristics and maternal smoking and alcohol consumption. Data on maternal height and weight and on infants' birth weight and gestational age were taken from hospital records.

**Results:**

Birth weight and gestational age of Roma infants was 373 (SE 15) g and 0.92 (0.05) weeks, respectively, lower than in non-Roma infants. Controlling for demographic, socioeconomic and behavioural factors reduced these differences to 133 (18) g and 0.57 (0.06) weeks, respectively (all p-values < 0.001). In terms of binary outcomes, the Roma vs. non-Roma odds ratios were 4.5 (95% CI 3.7–5.4) for low birth weight (< 2500 g), 2.8 (2.2–3.4) for preterm birth (< 37 weeks of gestation), and 2.9 (2.5–3.4) for intrauterine grown retardation (<10^th ^percentile of birth weight for gestational age); controlling for all covariates reduced these odds ratios to 1.7 (1.3–2.2), 1.5 (1.1–2.0) and 1.3 (1.0–1.6), respectively. Maternal education made the largest contribution to the ethnic differences; the role of health behaviours was relatively modest.

**Conclusion:**

There are striking differences in birth outcomes between Roma and non-Roma mothers. The causes of these differences are complex but largely socioeconomic.

## Background

The Roma people (Romanies, Gypsies, of northern Indian origin) are the most important ethnic minority in Central and Eastern Europe. It has been known for a long time that the socioeconomic conditions of most Roma people in Central and Eastern Europe are worse than those of the general population, and it has been suspected that their health follows a similar pattern. However, the main conclusion of two recent reviews was that there is a striking lack of information about the Roma people [1,2]; even the estimates of the size of their population vary widely [1], and data on their health are sparse and incomplete [1,2].

Available data suggest that the health of the Roma people is poor [1,2]. Similarly to ethnic differences in other countries [3], the contribution of different factors, such as culture, genes, behaviours or socioeconomic status, remains unclear. One particular area of great interest is reproductive health and birth outcomes. Data from Bulgaria, Hungary, Czech Republic and Slovakia indicate that abortions, low birth weight and premature birth are more common in Roma than non-Roma populations [4-9]. Given the problems with obtaining funding for such research and the difficulties with recruiting the Roma people into epidemiological studies, virtually all studies so far were of small size and most were conducted in non-representative population samples.

In this paper, we analysed a large population based study of births in two Czech communities. The study was originally designed to investigate the effect of air pollution on birth outcomes [10] but it also collected data on ethnicity and health behaviours known to be associated reproductive outcomes. Preliminary results from the first year of the study indicated that ethnicity was an important determinant of birth weight, gestational age and foetal growth [5]. Here we report the analyses of 10-year birth series. The paper has two objectives. First, to quantify the differences between Roma and non-Roma (i.e. European origin) infants in birth weight, gestational age and intrauterine growth. The second objective was to estimate the contribution of demographic variables, socioeconomic disadvantage and health behaviours to the ethnic differences.

## Methods

### Setting and subjects

As mentioned above, the study was set up to examine the effects of air pollution [10]. It was conducted in two Czech districts: Teplice (North Bohemia, high levels of air pollution) and Prachatice (South Bohemia, clean air). All singleton births from April 1995 through 2004 to women with at least 1 year of residence in the district were included in the study; exclusions due to short-term residence were very rare and cannot not bias the study. Virtually all deliveries in these districts during the study period were hospitalised, and women were enrolled during their stay in hospital. Given the almost universal hospitalisation of deliveries and the low refusal rate (less than 5% in each district), the births recruited into the study are representative for the two communities. Multiple births were excluded.

### Measurements

During hospitalisation, women completed a self-administered questionnaire with a help of a specially trained nurse. Ethnicity was based on maternal self-report; the dataset contained the following options: Asian; European; Roma, and "other".

Mother reported their highest attained education (primary or less; apprenticeship; secondary; university), marital status (married; cohabiting; single; divorced/widowed), smoking (cigarettes per day) and consumption of alcohol (drinks per week) before and during pregnancy. Data on mothers' height (in cm), pre-pregnancy weight (in kg), birth weight (in grams) and gestational age (in weeks) were taken from hospital records. The study attempted to collect data on fathers but we did not use these data because of the high rate of missing values (> 60% of values in variables related to father were missing).

### Statistical analysis

Because the numbers of Asian and "other" infants were too small for meaningful analyses (84 and 62 births, respectively), the final dataset was restricted to European and Roma babies. The statistical analysis was conducted in several steps. First, socioeconomic and life style variables were tabulated by ethnicity. Second, we compared crude differences between the two ethnic groups in the following birth outcomes: birth weight and gestational age measured as continuous variables, and low birth weight (< 2500 g), preterm birth (gestational age < 37 weeks), and intrauterine growth retardation (IUGR, < 10^th ^gender-specific percentile of birth weight for gestational age) defined as binary variables. Finally, we examined the extent to which the differences between Roma and non-Roma birth outcomes can be explained by available covariates. The statistical models (for both binary and continuous outcomes) were built as follows. First, crude effects of ethnicity were estimated. Second, the ethnic differences were adjusted separately for: (i) demographic characteristics of the infant (gender and district of birth); (ii) maternal demographic characteristics (maternal age and number of pregnancies); (iii) maternal social characteristics (education and marital status); (iv) maternal size (height and weight); and (v) maternal behaviours (smoking and alcohol consumption). Finally, all these covariates were entered into one model. All analyses were conducted using the STATA statistical software (Stata Corporation, College Station, Texas, USA).

## Results

The final dataset consisted of 10,326 singleton births, of whom 1388 (13%) occurred among Roma women. The distribution of socioeconomic characteristics and behaviours is shown in table 1. The high proportion of Roma infants in Teplice reflects the ethnic composition of the two districts. Roma mothers (and infants) had much less favourable profile in most characteristics, except of body mass index and alcohol consumption. Roma infants had considerably lower birth weight, somewhat shorter gestation, and much higher rate of IUGR (table 2).

**Table 1:** Descriptive characteristics of the infants/mothers.

	Non-Roma (n = 8938)	Roma (n = 1388)	p-value
District			
Teplice	77%	94%	< 0.001
Prachatice	22%	6%	

Male sex (%)	51%	51%	0.995

Number of pregnancies (%)			
1	37%	27%	< 0.001
2	30%	22%	
3+	33%	51%	

Maternal age group (%)			
< 19	7%	21%	< 0.001
20–24	38%	38%	
25–29	34%	24%	
30–34	15%	10%	
35+	5%	6	

Marital status (%)			
Married	72%	29%	< 0.001
Cohabiting	11%	26%	
Single	12%	41%	
Divorced/widowed	5%	4%	

Education (%)			
Primary or less	15%	81%	< 0.001
Apprenticeship	46%	17%	
Secondary	33%	2%	
University	6%	0%	

Maternal height, cm, mean (SD)	166.2 (6.4)	159.4 (7.0)	< 0.001

Maternal weight, kg, mean (SD)	62.7 (12.2)	56.7 (11.5)	< 0.001

Maternal body mass index, kg/m2, mean (SD)	22.6 (4.0)	22.4 (4.3)	0.01

Maternal smoking (%)			
Ever	42%	78%	< 0.001
Before pregnancy	35%	73%	< 0.001
During 1^st ^trimester	25%	67%	< 0.001
During 2^nd ^trimester	18%	65%	< 0.001
During 3^rd ^trimester	16%	63%	< 0.001

Maternal alcohol consumption, any (%)			
Before pregnancy	48%	31%	< 0.001
During pregnancy	22%	21%	0.491

**Table 2:** Birth outcomes in the Roma and non-Roma infants.

	Non-Roma (n = 8938)	Roma (n = 1388)	p-value
Birth weight, g, mean (SD)	33442 (483)	2970 (522)	< 0.001
Gestational age, weeks, mean (SD)	39.6 (1.5)	38.7 (2.0)	< 0.001
			
Low birth weight (< 2500 g) (%)	3.6%	14.1%	< 0.001
Preterm birth (< 37 weeks) (%)	3.9%	9.9%	< 0.001
IUGR (< 10^th ^percentile)(%)	8.9%	22.2%	< 0.001

Table 3 shows the extent to which different characteristics explain differences between Roma and non-Roma infants in birth weight and gestational age. In crude analyses, Roma infants were 373 g lighter at birth and their gestational age was 0.92 weeks shorter than of non-Roma babies. Gender, district, maternal age and number of pregnancies contributed only marginally to these differences. In contrast, maternal education "explained" more than one third of the difference in birth weight and more than one fifth of the difference in gestational age (the role of marital status was only marginal). Maternal size, and to a lesser extent smoking, also accounted for some of the ethnic differences. After controlling for all covariates available, the ethnic differences in birth weight were reduced by almost two thirds (from 373 g to 133 g); for gestational age, the reduction was smaller, about 40% (from 0.92 weeks to 0.57 weeks).

**Table 3:** Explaining ethnic differences in birth weight (g) and gestational age (weeks): results of linear regression (differences between Roma and non-Roma infants, standard errors and p-values).

**Adjusted for**	**Birth weight**			**Gestational age**		
	
	**Diff.**	**(SE)**	**p-value**	**Diff.**	**(SE)**	**p-value**
Crude	-373	(14)	< 0.001	-0.92	(0.05)	< 0.001
Infants' demographic characteristics (gender and district)	-363	(14)	< 0.001	-0.89	(0.05)	< 0.001
Mothers' demographic characteristics (maternal age, number of pregnancies)	-354	(15)	< 0.001	-0.87	(0.05)	< 0.001
Mothers' social characteristics (education, marital status)	-237	(17)	< 0.001	-0.73	(0.06)	< 0.001
Maternal height and weight	-260	(15)	< 0.001	-0.80	(0.05)	< 0.001
Maternal smoking and alcohol consumption	-292	(16)	< 0.001	-0.81	(0.05)	< 0.001
**All covariates listed above**	**-133**	**(18)**	**< 0.001**	**-0.57**	**(0.06)**	**< 0.001**

Results of logistic regression for binary outcomes were similar (table 4). For low birth weight, the crude odds ratio of 4.5 was reduced to 1.7 in the full model; for preterm birth, the odds ratio 2.8 fell to 1.5 after controlling for all covariates; and for IUGR, the covariates explained most of the excess in Roma infants. For all outcomes, education made the largest contribution to the reduction of the crude odds ratios.

**Table 4:** Explaining ethnic differences in low birth weight, preterm birth and IUGR: results of logistic regression (odds ratios for Roma vs non-Roma, 95% confidence intervals and p-values).

**Adjusted for:**	**Low birth weight**			**Preterm birth**			**IUGR**		
	
	**OR**	**95% CI**	**p-value**	**OR**	**95% CI**	**p-value**	**OR**	**95% CI**	**p-value**
Crude	4.5	(3.7–5.4)	< 0.001	2.8	(2.2–3.4)	< 0.001	2.9	(2.5–3.4)	< 0.001
Infants' demographic characteristics (gender and district)	4.3	(3.5–5.2)	< 0.001	2.5	(2.1–3.1)	< 0.001	2.9	(2.5–3.3)	< 0.001
Mothers' demographic characteristics (age, number of pregnancies)	3.9	(3.2–4.8)	< 0.001	2.4	(1.9–3.0)	< 0.001	2.8	(2.4–3.3)	< 0.001
Mothers' social characteristics (education, marital status)	2.6	(2.0–3.3)	< 0.001	2.0	(1.5–2.6)	< 0.001	1.8	(1.5–2.1)	< 0.001
Maternal height and weight	2.9	(2.4–3.6)	< 0.001	2.2	(1.8–2.8)	< 0.001	2.0	(1.7–2.3)	< 0.001
Maternal smoking and alcohol consumption	3.3	(2.6–4.1)	< 0.001	2.1	(1.7–2.7)	< 0.001	2.2	(1.9–2.6)	< 0.001
**All covariates** **listed above**	**1.7**	**(1.3–2.2)**	**< 0.001**	**1.5**	**(1.1–2.0)**	**0.010**	**1.3**	**(1.0–1.6)**	**0.029**

In additional analyses, we found no evidence for an interaction between ethnicity and calendar year: the differences between Roma and non-Roma groups remained constant over the study period (not shown in table). There was also no interaction between ethnicity and district; despite the difference in the size of the Roma population between the two districts, the differences between Roma and non-Roma groups were similar in Teplice and in Prachatice (not shown in table).

## Discussion

This study in a representative community sample of births found striking ethnic differences in all birth outcomes studied and in most socio-demographic characteristics. The covariates available in the study, particularly maternal education, explained a considerable part of these differences.

Several limitations of the study should be considered when interpreting the results. First, more detailed data would provide a more complete picture of the family environment. It is likely that if information on economic and material conditions (e.g. income, housing, household amenities) would contribute further to the explanation of the ethnic differences. Data on husbands would also help but, as explained above, the proportion of missing data on fathers was too high to be used in the analyses. Data on household assets would also be valuable to improve the assessment of families' socioeconomic status. Similarly, data on factors that could mediate the link between ethnicity, socioeconomic characteristics and birth outcomes, such as nutrition, would be also useful. Finally, ethnic differentials in birth outcomes may be further exacerbated by unmeasured area-level characteristics [11].

Second, some of the measurements might have been inaccurate. In particular, the definition of ethnicity is a difficult issue. Some misclassification is likely; some Roma mothers may have identified themselves as non-Roma. However, self-reported ethnicity, used in this study, has been suggested as the preferred method [3], and alternative methods are not ideal either. In addition, mothers may have misreported some of the information (e.g. smoking or alcohol consumption). Birth outcome variables were taken from medical records; while in a few cases these variables may have been recorded inaccurately by the hospital personnel, the validity of such information in this study is high [12]. In addition, assuming that such misclassification was random, it would tend to underestimate the associations studied, rather than lead to spurious findings.

Finally, the number of Roma births was much smaller than that of non-Roma births, and the unbalanced structure of the sample might have reduced the statistical power. However, given the overall size of the study, the statistical power was sufficient to demonstrate, in Roma vs. non-Roma comparisons, an odds ratio of 1.20 or higher at 95% confidence level. The probability of the beta error is therefore low.

In the research on ethnic differences in health in general, an important questions has been debated for some time, namely what are the reasons for such differences [3]. For different health outcomes, the proposed explanations range from genetic to socioeconomic factors [13-17] but addressing this questions has been hampered by limitations of available data [3]. Our results shed new light at one specific type of ethnic differences in health – the poor birth outcomes of Roma mothers. It has been generally perceived that the poor health of Roma people is largely due to their unhealthy behaviours, such as smoking and drinking. Our results suggest that the explanation is more complex. Smoking before and during pregnancy was considerably more common in Roma women but it statistically explained a relatively modest part of the excess of poor birth outcomes of Roma mothers. Alcohol consumption, on the other hand, was not more common in Roma mothers, and cannot therefore be implicated in their poor pregnancy outcomes.

Maternal education made by far the largest single contribution to explaining the poor birth outcomes in Roma mothers. This is not surprising, because there were huge differences in educational attainment between Roma and non-Roma mothers in this study (table 1), and because maternal education has been previously shown to be the key determinant of low birth weight, preterm birth, intrauterine growth and infant mortality in the Czech population [18,19]. In this study, the crude differences in mean birth weight between infants born to mothers with primary and university education was 322 g. It is therefore entirely plausible that education plays an essential part in the differences between the two ethnicities, not least because maternal education is a good proxy for a variety of measures of deprivation. Marital status was also strongly associated with birth outcomes (e.g. the crude differences in birth weight between married and single mothers was 232 g) but its contribution to the ethnic differences was smaller than that of education.

Maternal size, particularly height, partly reflects socioeconomic conditions in earlier life [20]. Reproductive history, indicated by the number of pregnancies, is also associated with social status. It is therefore likely that the ethnic differences in birth outcome in the Czech Republic are to a considerable extent determined by socioeconomic factors. This is consistent with the conclusions of a recent review of the literature on the ethnic differences in health in United States and Britain [3]. It would be interesting to explore whether factors such as nutrition or use, access to and quality of antenatal care can help further clarify the pathways linking ethnicity, socioeconomic circumstances and health.

## Competing interests

The author(s) declare that they have no competing interests.

## Authors' contributions

MB analysed the data and drafted the paper. JD and RJS designed and coordinated the study and commented on the drafts of the paper. IS contributed to the data collection and data management and commented on the drafts of the paper.

## Pre-publication history

The pre-publication history for this paper can be accessed here:


